# Subsequent MRI of pediatric patients after an adverse reaction to Gadolinium-based contrast agents

**DOI:** 10.1371/journal.pone.0230781

**Published:** 2020-04-03

**Authors:** Azadeh Hojreh, Andreas Peyrl, Aleksandra Bundalo, Zsolt Szepfalusi

**Affiliations:** 1 Department of Biomedical Imaging and Image-guided Therapy, Medical University of Vienna, Vienna, Austria; 2 Department of Pediatrics and Adolescent Medicine, Medical University of Vienna, Vienna, Austria; McLean Hospital, UNITED STATES

## Abstract

**Background:**

Gadolinium-based contrast agent (GBCA)-enhanced magnetic resonance imaging (MRI) scans often must be used repeatedly in pediatric oncologic patients. Although GBCAs are usually well tolerated, severe and life-threatening allergic reactions might occur, which can result in overly cautions adherence to special precautions in patients.

**Purpose:**

To evaluate the management of the reported GBCA-associated adverse reactions in subsequent contrast-enhanced MRIs in pediatric patients, distinguishing non-allergic and allergic reactions.

**Materials and methods:**

In this retrospective, cross-sectional study, consecutive pediatric neurooncological patients who underwent GBCA-enhanced MRI at our university hospital, between 2007 and 2016, were eligible. The patients’ history was evaluated with regard to any adverse events after GBCA administration. In a subset of patients with reported adverse reactions, the institutional premedication regime or an allergy work-up in clinical practice were performed, using either skin-prick tests or intravenous provocation tests in a double-blind procedure.

**Results:**

Included were 8156 contrast-enhanced MRI scans in 2109 patients. Nineteen acute adverse events (19/8156; 0.23%) in 17 patients (17/2109; 0.81%) were reported. Despite a premedication regime in 14 patients, three patients (3/14; 21.4%) reported a breakthrough reaction. None of the 12 patients who underwent skin-prick tests or intravenous provocation tests showed allergic reactions. At least one well-tolerated GBCA was identified in almost every tested patient.

**Conclusion:**

A fast-track allergy work-up can help to distinguish non-allergic and allergic reactions and to identify a well-tolerated GBCA, thus avoiding unnecessary premedication for subsequent GBCA administrations.

## Introduction

Repeated Gadolinium-based contrast agent (GBCA)-enhanced Magnetic resonance imaging (MRI) examinations are frequently required in the diagnosis and follow-up of pediatric patients, especially in patients with central nervous system (CNS) tumors, which constitute the largest group of solid neoplasms in children [1, 2].

GBCAs have been approved for parenteral use since the late 1980s and are extremely well tolerated by the vast majority of patients [3]. The incidence of acute adverse reactions is lower than that observed after the administration of iodinated contrast media [3]. Such reactions can be categorized as either allergic-like hypersensitivity or chemotoxic responses [4], and are classified either into four grades according to the Ring and Messmer classification [5], or into three categories of severity (mild, moderate, or severe) according to the American College of Radiology (ACR) Committee on Drugs and Contrast Media [3] or according to the European Society of Urogenital Radiology (ESUR) guidelines on contrast agents [4].

At the usually administrated clinical doses, adverse reaction rates are rare, ranging from 0.07% to 2.4% [3]. Most reactions are mild, including coldness, warmth, pain at the injection site, nausea with or without vomiting, headache, paraesthesia, and dizziness [3]. Allergic-like reactions are even less frequent, with an incidence of 0.004% to 0.7%, and severe life-threatening anaphylactic reactions are extremely rare (0.001% to 0.01%) [3].

Previously reported adverse reactions of patients to GBCA can be a serious clinical problem for future examinations due to the risk of an allergic-like reaction. Identifying patients at risk for allergic reactions is of the utmost importance in order to reduce the prevalence of allergic events to as close to zero as possible. The frequency of acute adverse reactions to GBCA is higher in patients with a previous reaction to GBCA [3, 6]. Corticosteroid and antihistamine premedication prior to contrast-enhanced studies that utilize a similar contrast material have not shown sufficient clinical effects, and so-called “breakthrough reactions” still occur [3, 7, 8] and have been controversially discussed in the guidelines [3, 4]. Moreover, the most commonly used premedication regimes are time-consuming. According to the Lasser scheme, the first medication is applied between six and 24 hours before and the second medication two hours before the contrast agent administration [9], and, according to the Greenberger scheme, the first medication should be administered 13 hours before, the second medication seven hours before, and the third medication one hour before the contrast agent application [10], which means additional effort for patients and unnecessarily high costs [7, 11].

In the case of contrast agent adverse events, the ESUR suggests, using a different contrast agent in subsequent examinations after consultation with a specialist in drug allergies, and does not recommend a premedication [4]. The usefulness of a skin-prick test and an intradermal skin test with contrast media to predict the likelihood of adverse reactions remains controversial [3, 12–14]. Moreover, the allergy label often persists despite a negative skin test and patients continue to receive premedication [15, 16]. Drug provocation tests are considered the gold standard for the diagnosis of an allergy to non-steroidal anti-inflammatory drugs (NSAIDS), local anesthetics, non-beta lactam antibiotics, and other drugs for which safer tests do not exist or are not standardized [17].

To our knowledge, there are two reported experiences about intravenous provocation testing to rule out GBCA-associated allergic reactions [18] and iodine contrast agent-associated allergic reactions in adult patients [19].

The purpose of this retrospective study was to evaluate the management of the reported GBCA-associated adverse reactions in subsequent contrast-enhanced MRIs in our pediatric center, distinguishing non-allergic and allergic reactions.

## Materials and methods

The institutional review board of Medical University of Vienna approved the study and waived the requirement for informed patients’ and parental consent, because the study was a retrospective data analysis. (IRB 1321/2018). All procedures performed in the study that involved human participants were in accordance with the ethical standards of the institutional review board and with the 1964 Helsinki declaration and its later amendments.

All patients of the Department of Pediatrics and Adolescent Medicine with intracranial lesions, who underwent an MRI scan in the Department of Biomedical Imaging and Image-guided Therapy between January 2007 and December 2016, were reviewed retrospectively. Patients with GBCA-enhanced MRI scans were evaluated with regard to the presence or absence of adverse reactions and their severity according to the ACR and the ESUR guideline classification system, and reactions were categorized as mild, moderate, or severe [3, 4].

Mild reactions included mild urticaria, mild itching, erythema, nausea, mild vomiting, warmth, chills, anxiety and vasovagal reaction, which resolved spontaneously [3, 4]. Moderate reactions included marked urticaria, mild bronchospasm, facial and laryngeal edema, and a moderate vasovagal reaction [3, 4]. Severe reactions included hypotensive shock, respiratory arrest, cardiac arrest, arrhythmia, and convulsions [3, 4].

In the case of a reported GBCA-associated adverse reaction, there are three methods in our pediatric center with which to manage the subsequent MRI: either the institutional pediatric premedication regime, modified according to Greenberger et al. [3, 10], or one of the two allergy work-ups, which are used in clinical practice, either by skin prick testing or by intravenous provocation tests.

### Institutional pediatric premedication regime

Our institutional premedication guidelines for the prevention of allergic-like reactions in children include a combination of intravenous prednisone 0.5–0.7 mg/kg (up to 50 mg) and intravenous diphenhydramine 1.25 mg/kg (up to 50 mg) one hour prior to contrast agent injection [3, 10].

### Skin-prick test

Skin testing was typically performed with a set of four GBCAs (Gadoterate meglumine, Gadobutrol, Gadoteridol, and Gadobenate dimeglumine) for optimal evaluation of potential cross-reactivity and identification of alternatives. Briefly, skin-prick tests were performed with the undiluted, commercially available solution [14]. Evaluation for reactions to GBCAs was performed 15 minutes after skin-prick tests, and, for non-immediate hypersensitivity reaction, there was a delayed reading of the skin-prick tests. The immediate-reading skin-prick test was considered positive if the size of the wheal was at least 3 mm in diameter, with surrounding erythema after 15 minutes. Immediate and optional delayed reading of skin-prick tests was performed according to the international guidelines of the European Society of Contact Dermatitis [20].

### Intravenous provocation test

Patients were provoked in a double-blind procedure with two different intravenous GBCAs on two different days, each preceded or followed by a placebo. Patients were informed that they would receive placebos, but were not told when they would be administered. The intravenous provocation was performed with the routinely administered doses of each GBCA. All provocation tests were performed under strict hospital surveillance, with emergency room facilities equipped to handle any anaphylactic reactions.

Patients were observed for at least 120 minutes after the last intravenous administration. Vital signs and physical examination were performed at baseline and every 30 minutes. If a reaction occurred, vital signs and physical examination were repeated.

The adverse reactions were classified according to the recommendation of the ESUR guidelines on contrast agents into hypersensitivity/allergy-like reactions, including cutaneous, respiratory and cardiac reactions, or chemotoxic/non-allergic reactions, including gastrointestinal and vasovagal reactions, which resolved spontaneously with no changes in vital signs, cardiac arrhythmia or convulsions [3, 4].

### Statistics

Descriptive statistics only were used for this study.

## Results

During the study period, 2109 patients with intracranial lesions underwent 9825 MRI scans (1158 males and 951 females; mean age, 7.86±5.91 years).

In total, 8156 GBCA-enhanced MRI examinations and 1669 non-contrast MRIs were performed during this observation time (Table 1).

**Table 1:** Gadolinium-based contrast agents applied during MRI scans in the evaluated population.

Gadolinium-based Contrast Agent	No. of MRI scans
**Gadoterate meglumine**	6530
**Gadobutrol**	512
**Gadobenate dimeglumine**	425
**Gadoteridol**	300
**Gadoxetate disodium**	28
**Gadodiamide**	4
**GBCA, not otherwise specified**	357

In our collective, acute adverse reactions were reported in 17 patients (17/2109; 0.81%; 11 males and six females, mean age, 10 ± 4.47 years; range, 3–17 years at the first adverse reaction), either by the patients, parents, or radiologists. Two of these patients (patients 2 and 5) reported two acute adverse reactions after two different GBCAs (Table 2). In total, there were 19 acute adverse reactions reported after 8156 GBCA i.v. administrations (0.23%).

**Table 2:** Patients with a reported adverse reaction to a Gadolinium-based contrast agent.

No of Patient	Sex	Diagnosis	No. of MRIs with GBCA before the first AR	Age at the first AR	GBCA at the AR	Reported symptoms	Grade (Ring & Messer classification)	Classification of AR
**1**	m	Atypical teratoid rhabdoid tumor	22	7	Gadoterate meglumine	Urticaria	1	Mild
**2**	f	Pilomyxoid astrocytoma	22	7	Gadoterate meglumine	Exanthem all over the body, face wheals, coughing, acute respiratory insufficiency	2	Moderate
					Gadoteridol			
**3**	m	Endo- and suprasellar isomorphic pilocytic astrocytoma	49	15	Gadoterate meglumine	Heat sensation, nausea, chills, tachycardia, face erythema	1	Mild
**4**	f	Sella turcica tumor	4	16	Gadoterate meglumine	Nausea, vomiting, collapse	1	Mild
**5**	m	Atypical papillary glioneuronal tumor	0	12	Gadoterate meglumine	Itching, rash, neck wheals	1	Mild
					Gadobutrol			
**6**	f	Pilocytic astrocytoma	10	5	Gadobenate dimeglumine	Tonic-clonic convulsion, intermittent respiratory complaints	4	Severe
**7**	f	Pilocytic astrocytoma	3	4	Gadoterate meglumine	Flush, erythema	1	Mild
**8**	m	Tumor of unknown origin in pons	0	3	Gadoterate meglumine	Vomiting	1	Mild
**9**	m	Diffuse infiltrative isomorphic oligoastrocytoma	10	17	Gadoterate meglumine	Nausea, dizziness	1	Mild
**10**	f	Suprasellar germinoma	6	9	Gadoterate meglumine	Vomiting, chills, red spots on the neck	1	Mild
**11**	m	Optic pathway glioma	9	9	Gadoterate meglumine	Nausea, vomiting	1	Mild
**12**	m	Cerebellopontine angle mass	8	13	Gadoterate meglumine	Headache, nausea, vomiting	1	Mild
**13**	f	Pilocytic astrocytoma	0	12	GBCA, nos	Local swelling	1	Mild
**14**	m	Pilocytic astrocytoma	5	7	Gadoterate meglumine	Vomiting, flush	1	Mild
**15**	m	Hyperprolactinemia without a tumor	0	17	Gadoteridol	Parasternal urticaria	1	Mild
**16**	m	Dysplastic ganglioglioma	13	9	Gadoterate meglumine	Generalized tonic-clonic convulsion	4	Severe
**17**	m	Tectum glioma	0	12	Gadobenate dimeglumine	Nausea	1	Mild

GBCA, Gadolinium-based contrast agent; AR, Adverse reaction; nos, Not otherwise specified.

Thirteen acute adverse reactions were reported after Gadoterate meglumine administration (13/6530; 0.2%), two adverse reactions after Gadoteridol administration (2/300; 0.67%), two adverse reaction after Gadobenate dimeglumine administration (1/425; 0.24%), and one adverse reaction after Gadobutrol administration (1/512; 0.20%). In one case with a reported adverse reaction, the administered GBCA was not documented.

Sixteen reported acute adverse reactions (16/8156; 0.2%) were classified as mild (flushing, nausea, vomiting, urticaria), one (1/8156; 0.01%) as moderate (intermittent respiratory complaints), and two (2/8156; 0.02%) as severe (convulsions), according to the ACR and ESUR guidelines on contrast agents.[3, 4]

Fourteen of these patients received a premedication regime with corticosteroids and an antihistamine before subsequent GBCA administration. Despite the premedication regime, three patients (3/14; 21.4%) reported a breakthrough reaction (Table 3).

**Table 3:** Results of premedication and allergy tests in patients with reported adverse reactions to a Gadolinium-based contrast agent.

No of Patient	Sex	GBCA at the AR	Premedication	Breakthrough	Allergy Test	Results of skin-prick test (SPT)				Results of intravenous provocation test (IPT)			Identified well-tolerated GBCA	Re-exposure of a well-tolerated GBCA without premedication	No. of MRIs with well tolerated GBCA without premedication	AR after the re-exposure to a well-tolerated GBCA without premedication
						Gadoterate meglumine	Gadobutrol	Gadobenate dimeglumine	Gadoteridol	Gadoterate meglumine	Gadobutrol	Gadobenate dimeglumine				
**1**	m	Gadoterate meglumine	Yes	No	No	-	-	-	-	-	-	-	-	-	-	-
**2**	f	Gadoterate meglumine	Yes	Yes	**SPT**	Negative	Negative	Negative	Negative	-	-	-	All four GBCAs	No	-	-
		Gadoteridol														
**3**	m	Gadoterate meglumine	Yes	No	**IPT**	-	-	-	-	Dizziness & nausea	Dizziness & nausea	-	-	No	-	-
**4**	f	Gadoterate meglumine	Yes	No	No	-	-	—	-	-	-	-	-	-	-	-
**5**	m	Gadoterate meglumine	Yes	Yes	**IPT**	-	-	-	-	Dizziness & nausea & flush	Negative	Negative	Gadobenate dimeglumine, Gadobutrol	**Yes**	17	**No**
		Gadobutrol														
**6**	f	Gadobenate dimeglumine	No	Only non-contrast	**SPT IPT**	Negative	Negative	Negative	Hyper-salivation	Negative	Negative	-	Gadoterate meglumine; Gadobutrol	-	0*	-
**7**	f	Gadoterate meglumine	Yes	No	No	-	-	-	-	-		-	-	-	-	-
**8**	m	Gadoterate meglumine	Yes	No	**IPT**	-	**-**	**-**	**-**	Negative	Negative	-	Gadoterate meglumine; Gadobutrol	**Yes**	3	**No**
**9**	m	Gadoterate meglumine	Yes	No	**SPT**	Negative	Negative	Negative	Negative	-		-	All four GBCAs	**Yes**	4	**No**
**10**	f	Gadoterate meglumine	Yes	No	**IPT**	-	-	-	-	Vomiting	Negative	-	Gadobutrol	**Yes**	21	**No**
**11**	m	Gadoterate meglumine	Unknown	Unknown	No	-	-	-	-	-	-	-	-	-	-	-
**12**	m	Gadoterate meglumine	Yes	No	**IPT**	-	-	-	-	Negative	Negative	-	Gadoterate meglumine; Gadobutrol	**Yes**	3	**No**
**13**	f	GBCA, nos	Yes	Yes	**IPT**	-	-	-	-	Negative	Negative	-	Gadoterate meglumine; Gadobutrol	**Yes**	8	**No**
**14**	m	Gadoterate meglumine	Yes	No	**IPT**	-	-	-	-	Negative	Negative	-	Gadoterate meglumine; Gadobutrol	**Yes**	7	**No**
**15**	m	Gadoteridol	Yes	Unknown	No	-	-	-	-	-	-	-	-	-	-	-
**16**	m	Gadoterate meglumine	Unknown	Unknown	**IPT**	-	-	-	-	Negative	Negative	-	Gadoterate meglumine; Gadobutrol	**Yes**	5	**No**
**17**	m	Gadobenate dimeglumine	Yes	No	**IPT**	-	-	-	-	Negative	Negative	-	Gadoterate meglumine; Gadobutrol	**Yes**	2	**No**

* no scheduled MRI since allergy work-up; GBCA, Gadolinium-based contrast agent; AR, Adverse reaction; nos, Not otherwise specified; SPT, Skin-prick test; IPT, Intravenous provocation test.

Twelve patients underwent an allergy work-up. There were three patients with a breakthrough reaction, eight patients after a premedication regime without a breakthrough reaction, and one patient with no premedication (Table 3). The allergy work-up included skin-prick tests in three patients and intravenous provocation tests in ten patients (patient 6 had both tests). Two patients (patients 7 and 15) moved away, and the parents of three patients (patients 1, 4, and 11) refused an allergy work-up (Table 3).

Skin-prick tests in three patients for Gadoterate meglumine, Gadobutrol, Gadoteridol, and Gadobenate dimeglumine revealed skin-test negativity in all three patients, and in one of the patients a hypersalivation to Gadoteridol was reported (patient 6), which is a chemotoxic response, according to the ESUR guidelines [4]. Although the patients showed skin-test negativity, in clinical practice, only patient 9 had uneventful subsequent GBCA-enhanced MRIs without any premedication. Patient 2 still received premedication before GBCA administration and patient 6 underwent intravenous provocation tests (Table 3).

Twenty-one intravenous provocation tests were performed in ten patients for at least two substances—either Gadoterate meglumine, Gadobutrol, or Gadobenate dimeglumine (Table 3).

None of the patients who underwent intravenous provocation tests showed any sign of a hypersensitivity/allergic-like reaction. Two patients (patients 3 and 5) exhibited dizziness, nausea and flush, and one patient (patient 10) experienced vomiting (Table 3), which are all categorized as chemotoxic responses, but not as allergic-like reactions, according to European Society of Urogenital Radiology (ESUR) guidelines on contrast agents [4]. None of the patients needed any medication thereafter or showed signs of renal failure in follow-up examinations. Only in patient 3 were both tested GBCAs not well tolerated. In all other provoked patients, at least one well-tolerated GBCA without any reaction could be identified. All but one of the provoked patients had uneventful subsequent GBCA-enhanced MRIs without any premedication.

## Discussion

In our patient collective with reported GBCA-associated adverse events, there was no confirmed allergic reaction to GBCA, either with skin-prick tests or with intravenous provocation tests, although, the 0.23% incidence of GBCA-associated adverse events in our study was within the range of the published incidences in earlier pediatric studies, where an adverse reaction frequency of 0.04% - 19.3% was reported [21–23]. The administration of the well-tolerated GBCA identified by intravenous provocation tests or skin-prick tests in subsequent MRI examinations was uneventful.

Repeated GBCA administration is necessary in several clinical indications, including in the diagnosis and follow-up of pediatric oncological patients. Acute, life-threatening adverse reactions to GBCAs are rare, but have occurred, even though GBCAs are well tolerated by most patients [22–25].

A substantial number of patients with a history of prior reaction to GBCAs require additional contrast-enhanced examinations, which would expose them to the same or a similar contrast agent [8]. Self-reported allergy is always subjective, and, in many cases, inaccurate [26, 27]. Nevertheless, a suspected allergy to contrast media might escalate into a life-threatening anaphylactic event [28], leading to uncertainty in radiological departments, which usually establish special precautions to deal with these patients.

In patients with a previous adverse reaction to GBCA, the risk of a repeated adverse event is increased [3, 4, 6]. In many institutions, prophylactic premedication with corticosteroids and antihistamines is administered, to reduce the risk of allergic-like reactions in patients with a history of previous adverse events [3, 9]. However, adverse events to GBCA have been reported to occur despite premedication with corticosteroids and antihistamines, the so-called “breakthrough reactions” [7, 8]. In our series, we observed breakthrough reactions in 21.4% of the patients after premedication, which required further evaluation.

When an allergic-like reaction is suspected, skin testing may be useful to confirm an allergy and to identify alternative agents for subsequent studies [29]. Skin-testing revealed an excellent negative predictive value in patients with suspected GBCA hypersensitivity [30]. Although skin-testing can identify safe alternatives for GBCA re-exposure, and potentially discriminate between allergic and non-allergic reactions, the interpretations of the allergist, the radiologist, and the patient are often not well aligned [15]. Similar work in patients with penicillin allergy indicates that the allergy label often persists despite a negative work-up [16]. Patients with negative skin test results continued to avoid contrast media, mainly because of personal concerns or the radiologist's concerns [15]. One reason might be that skin tests do not reflect the real-life situation, as we also observed in two patients who refused a GBCA-enhanced subsequent MRI, even though no allergic reaction was confirmed by skin testing.

The European Academy of Allergy and Clinical Immunology Executive Committee considers drug provocation tests the gold standard for the diagnosis of an allergy to drugs [17]. In our study, the intravenous provocation testing was performed in ten patients under strict hospital surveillance with emergency room facilities. The intravenous provocation tests were well tolerated by all ten patients. None of the provoked patients showed symptoms attributable to an allergic reaction. Three patients reported dizziness, nausea, or vomiting, which were classified as clinically mild chemotoxic, non-allergic adverse reactions. We were able to identify at least one GBCA in nine of ten provoked patients that caused no reaction at all using intravenous provocation tests. All but one of the provoked patients had an uneventful, contrast-enhanced subsequent MRI with the well-tolerated GBCA identified by the intravenous provocation test. Based on our experience, the intravenous provocation test led to a remarkable reduction of concerns in patients, parents and radiologists, probably due to the better simulation of the real-life situation and uneventful subsequent MRI examinations with a tested GBCA. In a recent study, the experience with an intravenous provocation test with which to rule out a GBCA associated allergic reaction in adult patients was reported [18].

Because of the retrospective character of this study, our single-center study contains several limitations. The flow rate of the GBCA injection during the MR imaging (manually or via power injection) could not be verified, which has been discussed with regard to the appearance of acute dyspnea after i.v. administration of Gadoxetate disodium [31]. There was no blood sample collection within one hour after the acute adverse reaction [14]. Since reactions to Gadolinium are very rare, skin-prick tests and intravenous provocation tests could be performed in only a small sample size. Both tests, the skin-prick tests and the intravenous provocation tests were applied in one patient only; otherwise, the tests were performed in different patient groups, so these could not be compared in the same patients.

A prospective, multi-center study could verify our study results.

## Conclusion

In conclusion, a fast-track allergy work-up can help to distinguish non-allergic adverse reactions from allergic reactions in pediatric patients with reported GBCA-associated adverse events, and to identify a well-tolerated GBCA, thus avoiding unnecessary premedication for subsequent GBCA administrations.
